# Vaccination coverage trends in European Union from 1980 to 2020: A joinpoint Regression Analysis

**DOI:** 10.1093/eurpub/ckac129.306

**Published:** 2022-10-25

**Authors:** FA Causio, L Villani, M Mariani, R Pastorino, C De Waure, W Ricciardi, S Boccia

**Affiliations:** 1 Section of Hygiene, UCSC Rome, Rome, Italy; 2 Department of Medicine and Surgery, University of Perugia, Perugia, Italy; 3 Department of Woman, Child and Public Health, Fondazione Policlinico Universitario A. Gemelli IRCCS, Rome, Italy

## Abstract

Vaccinations are successful, cost-effective public health interventions; nevertheless, vaccine hesitancy represents a concern and several EU countries have implemented mandatory vaccinations to counteract it. The assessment of vaccination coverage data is helpful to clarify the reason behind this choice better and assess its impact. Data were extracted from the WUENIC database as of July 2021. All the 27 EU countries were included, considering the period from 1980 to 2020 (depending on data availability). Coverage indicators on seven vaccinations scheduled during the first year of life to prevent nine vaccine-preventable diseases were considered. Joinpoint regression was run using Joinpoint Trend Analysis Software 4.9.0.0. For each coverage indicator, the last two trends in time identified by the joinpoint regression were considered to identify countries with a positive vaccination coverage trend (having either the last trend significantly positive or the second last significantly negative but followed by a trend reversal) or a negative coverage trend (having either the last trend significantly negative or the second last significantly positive but followed by a trend reversal). To assess each country, we collated together information on each coverage indicator. A total of 180 jointpoint regressions were run. At least one joinpoint was observed in 144 cases: 39 (27.1%) showed a significant positive trend, and 49 (34%) had a significantly negative one. In 36 cases, there was a single trend lacking a joinpoint, either positive (21, 58.3%), negative (8, 22.2%), or not showing a change (7, 19.4%). Overall, 14 countries had mostly negative vaccination coverage trends, whereas 13 had mostly positive vaccination coverage trends. Systematised data collection and analysis of vaccination coverage trends are needed to support public health systems. EU countries differ broadly, but the overall situation shows that coverage trends are a key issue to be addressed.

**Key messages:**

• Vaccination coverage in the EU shows a general negative trend. The assessment and comparison of coverage trends across EU countries could make policymakers able to respond to critical issues timely.

• Mandatory vaccinations have been issued in different countries to prevent vaccination coverages from decreasing. Some countries have not issued any mandate but show high vaccination coverages.

